# Integrating postnatal care into the redesign of group care beyond birth

**DOI:** 10.1186/s13690-025-01508-4

**Published:** 2025-02-13

**Authors:** Ashley Gresh, Astrid Van Damme, Deborah L. Billings, Sharon Schindler Rising, Shaimaa Ibrahim, Abiola Ajibola, Ellen Chirwa, Jennyfer Don-Aki, Nastassia Donoho, Manodj Hindori, Nafisa Jiddawi, Emeka Kanebi, Esnath Kapito, Catherine Kay, Tara Kinra, Vlorian Molliqaj, Bolanle Oyeledun, Marlies E. B. Rijnders, Octavia Wiseman, Ghutai Sadeq Yaqubi, Crystal L. Patil

**Affiliations:** 1 Johns Hopkins School of Nursing, Baltimore, MD USA; 2 Vrije Universiteit Brussel, Jette, Belgium; 3 Group Care Global, Philadelphia, PA USA; 4 University of South Carolina, Columbia, SC USA; 5 United Nations Children’ Fund- Headquarters (UNICEF/ HQ), Nairobi, Kenya; 6 Centre for Integrated Health Programs (CIHP), Abuja, Nigeria; 7 Kamuzu University of Health Sciences, Blantyre, Malawi; 8 Jhpiego, Federal Capital Territory, Ankuru , Nigeria; 9 Partners in Health, Boston, MA USA; 10 Perisur Foundation for Perinatal Interventions and Research, Paramaribo, Suriname; 11 WAJAMAMA, Zanzibar, Tanzania; 12 Better Births Midwife, Princess Alexandra Hospital Trust and Hertfordshire and West Essex Local Maternity and Neonatal System, Hertfordshire, UK; 13 MIT World Peace University, Pune, India; 14 PLAY International, Pristina, Kosovo Kosovo; 15 TNO Child Health, Leiden, the Netherlands; 16 Centre for Maternal and Child Health Research, City, University of London, London, UK; 17 Jhpiego, Kabul, Afghanistan; 18 University of Michigan School of Nursing, Ann Arbor, MI USA

**Keywords:** Group care, Postnatal, Well-child, Health service delivery, Maternal and child health, Implementation science, CenteringParenting^®^

## Abstract

**Background:**

Globally, alarmingly high rates of maternal and infant mortality and morbidity persist. A constellation of health system and social factors contribute to this, including poor quality and barriers to accessing health care, including preventive services. As such, there have been calls for a redesign of maternal and child health (MCH) services. Although group care has primarily been tested in antenatal settings, it offers a promising redesign that optimizes maternal and child health care, survival, and well-being. The purpose of this study was to produce a blueprint of an adapted group care model that integrates postnatal maternal care, well-child care, and family engagement to be adapted to realities of different settings.

**Methods:**

Using a human-centered design approach and the Framework for Reporting Adaptations and Modifications to Evidence-based interventions (FRAME), we employed qualitative methods to adapt CenteringParenting^®^ (retaining its three core pillars of health assessment, interactive learning, and community building), and co-create the blueprint for group care beyond birth that can be used across settings. We initiated the process through face-to-face workshops during a global meeting on group care, followed by six online incubator sessions with key stakeholders from 13 countries during which we used qualitative methods of free listing, pile sorting, and ranking. We conducted a rapid qualitative analysis to produce a blueprint.

**Results:**

Participants collaboratively modified the content, format, and evaluation of CenteringParenting^®^ with the goal of creating a blueprint that integrates postnatal and pediatric care into group care that can be further adapted and implemented across diverse settings and contexts. The blueprint consists of suggested timing of visits over two years after birth, suggested visit content, and evaluation metrics for research and practice.

**Conclusions:**

The resulting group care beyond birth blueprint offers a strategy to redesign maternal and infant/child health services that can positively transform postnatal care and provide essential services to postpartum people. Adaptation of the blueprint to local realities is expected. Future research is recommended to test the model’s acceptability, feasibility, and effectiveness across settings. Using this blueprint, we can build the evidence base to support this model aiming to improve maternal and infant/child health outcomes.

**Supplementary Information:**

The online version contains supplementary material available at 10.1186/s13690-025-01508-4.

**Table:** 

Text box 1. Contributions to the literature
• This study addresses the need for extended, holistic postnatal care after birth by adapting the CenteringParenting model which was initially designed as a dyad model but in practice has primarily focused on the infant/child. Our adaptation aims to realize the model's full potential by providing comprehensive postnatal and pediatric care.
• The human-centered design process to produce the blueprint for the group care beyond birth model enhances its relevance and adaptability across different settings and cultural contexts.
• The group care beyond birth blueprint provides a guide to ensure the model is critically assessed and refined, contributing to continuous quality care improvement.

## Background

Despite progress made in maternal and child survival, high rates of maternal and infant mortality and morbidities persist globally [1]. Postpartum women and people who experience a maternal morbidity such as postnatal depression, uncontrolled hypertension, or infectious disease in the extended postnatal period (i.e., up to 12 months after birth) have lower quality of life and increased risk of mortality [2, 3]. Unaddressed postnatal morbidities (e.g., maternal depression and anxiety) can also directly impact the health and well-being of the infant, for whom the first year of life is a crucial time for their long-term health and development [4–6]. A constellation of health system and social factors contributes to maternal and infant/child morbidities, including low rates of postnatal visit attendance, dissatisfaction with care, poor quality care, inaccessible care, and inadequate preventive and promotive health care services [3, 7]. As such, there have been calls for a redesign of maternal and infant/child health services and delivery systems.

Group care models, inspired by CenteringPregnancy^®^ and CenteringParenting^®^, hold significant promise for transformative maternal and child health care redesign. These Centering-inspired group care models focus on the well-being of pregnant and postnatal people and infants/children so they can reach their full potential. Maternal and child health-related group care has been predominantly evaluated in antenatal settings. This growing body of rigorous evidence substantiates group care’s effectiveness for improving maternal and infant health-related outcomes including greater antenatal care attendance and satisfaction, more facility-based births, and improved health literacy outcomes [8–10]. Furthermore, selected studies demonstrate the feasibility of scaling group antenatal care in both high-income countries and low- and middle-income countries (LMICs). By increasing the utilization of health services, group care has the potential to improve both short-term and long-term health and well-being for parents and children and to promote equitable health care delivery, laying the foundation for healthier future generations in communities globally [11, 12]. Additionally, health care providers report that group care offers opportunities to deliver high-quality care that benefits women and families and allows them to further develop their professional role [13].

CenteringParenting^®^, a model of group care beyond birth that originated in the United States, brings together 6–8 postnatal people with similarly aged infants/children to engage in care as a group [14]. Each visit is 1.5 to 2 hours long with the first 30–45 minutes devoted to standard clinical health assessments for the infant/child and postnatal person and participation in self-assessments such as weight, blood pressure, and growth monitoring (e.g., weight and length) of their infant/child. Health assessments are followed by 75–90 minutes of facilitated, interactive health promotion activities. Group care facilitators are trained to foster participant-led discussions based on a planned yet flexible set of topics, highlighting issues most pertinent to postnatal individuals and their families.

The extension of this model to the postnatal period out to two years after birth provides an opportunity to address ongoing maternal and child morbidities and social determinants of health, while supporting the transition of the postpartum person-infant/child dyad from postnatal to primary care during this critical period in the life course. The World Health Organization (WHO) acknowledges the growing body of evidence that demonstrates the importance of considering the postnatal period beyond six weeks to up to one to two years after birth [15].

Preliminary evidence suggests that group well-child care in the United States is an efficient and family-centered model that influences clinical outcomes and has the potential to meet the needs of underserved populations [16]. However, few studies examine the longer-term effects of group care on both maternal and child health into the longer *postnatal* period of up to two years. The group care model and approach to health promotion and care aim to be responsive to the parent/child dyad’s needs and delivered in a holistic manner. However, current implementation predominantly focuses on the infant/child rather than also including the postnatal individual [17]. This is because substantial systemic changes are needed to offer integrated care for both the postnatal person and the infant/child. For example, beyond mental well-being, health assessments for postnatal individuals are not typically completed. Integrated group care for the dyad beyond the first six weeks after birth is not widely implemented. Adapting the current implementation of CenteringParenting^®^, which is now focused on pediatric care to provide care to both the postpartum person and their infants/children is needed to improve both maternal and child health, thereby improving the lives of families.

This study employed a human-centered design approach (HCD) to co-design an adapted Centering-based group care beyond birth model blueprint for use across countries and contexts. We define “blueprint” as a comprehensive plan outlining the goals and strategies of group care beyond birth as well as the inclusion of a timeframe, and relevant progress and performance indicators that can be tailored to each setting in which it is implemented [18]. Adaptation and modification are key concepts in implementation science because the process of implementing evidence-based practice is dynamic [19]. It is important to understand how, when, and why adaptations and/or modifications occur to strive for enhancing the interventions’ effectiveness while also maintaining fidelity [19]. To describe and systematically report the process of adapting and modifying CenteringParenting^®^ and to describe the resulting blueprint, this study uses the expanded Framework for Reporting Adaptations and Modifications to Evidence-based interventions (FRAME) [19]. The resulting blueprint provides the foundation to introduce, implement, and evaluate group care beyond birth in different country settings and in diverse contexts.

## Methods

### Study design

This qualitative study used an HCD approach [20] and conducted a rapid qualitative analysis [21] to develop a blueprint for group care beyond birth. Incubator sessions, comparable to focus groups but always with a focus on brainstorming and engagement in the adaptation process, were held with participants, and qualitative research methods were employed during sessions including, detailed field notes, free listing, pile sorting and ranking [22]. These methods, used in combination with FRAME to track all adaptations and modifications of CenteringParenting^®^, offered a rapid and systematic way of examining intracultural variations and grounded a process for consensus building, toward the development of the blueprint of an adapted group care beyond birth model.

### Sample and recruitment

The sample is comprised of professionals from around the world who attended the Global Group Care Catalyzer meeting in February 2024 in Nairobi, Kenya (a global event to discuss group care scale-up organized by Jhpiego and supported by the Gates Foundation). The study team asked participants from the Global Group Care Catalyzer event to identify key stakeholders to participate via word of mouth (snowball sampling). Through this process, other key stakeholders, including clinicians, researchers and group care implementers, participated in the study. Exclusion criteria included being under the age of 18 years old, non-English speaking, and adults lacking capacity to consent.

### Data collection

#### Description of workshops

This work was initiated at four workshops held during the Global Group Care Catalyzer meeting. Workshops were facilitated by two Catalyzer Planning Committee members and the main objectives were to define group care beyond birth and outline its key characteristics. Facilitators took detailed field notes and summarized information about the definition of group care beyond birth and its key characteristics during each workshop. To start the process of adapting the CenteringParenting^®^ model, workshop participants and other key stakeholders were invited to participate in virtual incubator sessions to co-create the blueprint of the group care beyond birth model after the Catalyzer.

#### Incubator session procedures

Virtual incubator sessions were held bi-monthly via Zoom at an agreed upon time by participants for three months (between March-May 2024). Times were scheduled to accommodate participants residing in multiple time zones. Each session was approximately one hour in length. Sessions were conducted until the group reached consensus about the timing and scheduling of visits and the session guide that retained the core pillars of the model -- health assessment in a group space, interactive learning, and community building. Participants also produced a suggested evaluation plan for research and practice focused on agreed upon outcomes and indicators. Facilitators recorded incubator sessions and stored them in a secure Zoom account. Recordings were made accessible to participants who were unable to attend a session so they could view the recording and provide input as the process was iterative with each session building on the previous one. Participants also had the option to provide input asynchronously via secure email.

The sessions began by asking participants to collectively free list areas they felt needed to be addressed in postnatal maternal and infant/well-child care. Free listing is a method to gather information on a topic by listing all the ideas participants can think of related to the topic being studied, in this case postnatal maternal and infant/well-child care [23]. An example of an open-ended question used was: “*What areas of health promotion are necessary to include in thepostnatal care visits*?” The participants were then asked to list topics for every area they could think of, and research leads recorded their responses. Facilitators provided additional explanations when necessary and prompted participants to ensure that they offered a comprehensive listing of ideas. The final step in this process was to reach consensus about which items to recommend for inclusion in the blueprint. To do this, each item was read aloud and through discussion the group decided to eliminate, merge, or keep the item.

Participants were then asked to review the final list of retained items and use pile sorting and ranking to prioritize items based on timing and appropriateness [24]. This process served two purposes. First, similar items were grouped. For example, for health assessments all items related to the cardiovascular health of the postnatal person were grouped together. Second, the grouped items for interactive learning topics were then aligned with the timing of recovery for the postnatal person and the age and stage of the child. For example, prioritizing topics to be covered at specific timepoints needed to reflect needs in the first week after birth versus six months after birth. Each item from the list created was written down and read aloud to participants. Through group discussions, participants drew on knowledge about their own context, practitioner experience, and existing guidelines and literature related to postnatal care to come to an agreement about when content should be introduced over the two years after birth. This sorting and ranking process led to the content areas that were included in the blueprint. This same consensus building process was used to generate a list of anticipated outcomes and indicators for researchers and practitioners to use for evaluation of the model.

### Data analysis

Incubator facilitators took detailed field notes during all sessions. These field notes did not contain identifiable personal information and focused on capturing participants’ ideas, concerns, feedback, and consensus about the blueprint of group care beyond birth. We then conducted a rapid qualitative analysis for development of the blueprint. Rapid qualitative analysis is recommended in implementation research so that preliminary results are turned around quickly and, in this case, are available for use to test the adapted model across settings [21]. Rapid qualitative analysis is primarily deductive drawing on a priori categories, but flexible enough to allow for new categories to emerge from the data. A priori categories were created based on the core components of the CenteringParenting^®^ model and the FRAME was a tool used to label adaptations. To ensure rigor, accuracy and validity we conducted member checking with all participants to finalize the blueprint [22].

This consensus process resulted in a blueprint of the group care beyond birth model that can be used as a guide to introduce, implement and evaluate it across countries and in diverse settings. In alignment with HCD and to enhance transparency in dissemination of the blueprint, some participants are co-authors on this manuscript. The team followed the consolidated criteria for reporting qualitative research (COREQ) [25].

### Ethics

This study received Institutional Review Board exemption from Johns Hopkins School of Medicine (IRB#00443165).

## Results

**Fig. 1:**
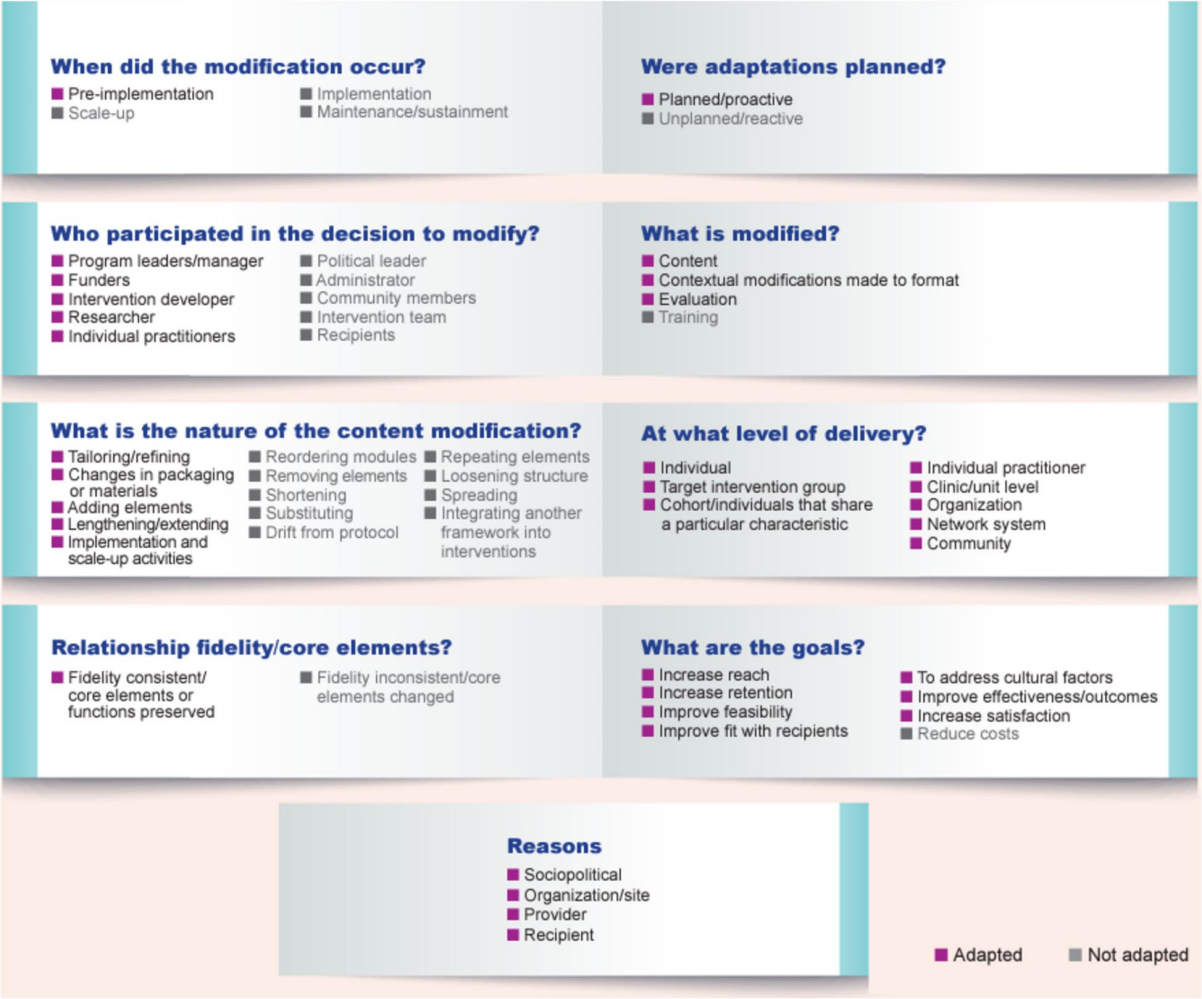
The adaptations and modifications made to CenteringParenting^®^ that led to the final group care beyond birth blueprint based on the FRAME categories

Four workshops and six incubator sessions were conducted with 25 people from 13 countries. Participants included clinicians, researchers, and implementers of group care, outlined in Table 1. We use the FRAME to guide descriptions and reporting of the adaptation process informing the blueprint (Fig. 1). The adaptation process ensured that the three core pillars of the original group care model were retained: health assessment (including self-assessment); interactive learning, and community building. Through the incubator sessions, as participants engaged in free listing, pile sorting, and ranking, participants came to a consensus about what to include in the adapted CenteringParenting^®^ blueprint of the dyad-focused group care model. We use the FRAME to describe what was included in the final blueprint, which consists of: a session guide for clinical care (see Fig. 1), interactive learning (see Figs. 1 and 3), and community building (see Fig. 1) as well as suggested evaluation metrics for both practice (see Table 2) and research (see Table 3).

**Table 1:** Participant characteristics

Participant	Country	Global Group Care Catalyzer workshop attendance	Role Related to Group Care
1	Afghanistan	Yes	Clinician, group care implementer
2	Belgium	No	Clinician, researcher
3	Haiti	Yes	Group care implementer, researcher, funder
4	India	Yes	Clinician, group care implementer
5	USA, working in Kenya	Yes	Program developer, researcher, funder
6	Kosovo	Yes	Group care implementer
7	Kosovo	Yes	Researcher
8	Malawi	Yes	Clinician, researcher, group care implementer
9	Malawi	Yes	Clinician, group care implementer, researcher
10	Malawi	Yes	Clinician, group care implementer
11	Nepal	Yes	Clinician, group care implementer
12	Netherlands	Yes	Clinician, group care implementer, researcher
13	Nigeria	Yes	Clinician, group care implementer
14	Nigeria	Yes	Clinician, group care implementer
15	Nigeria	Yes	Clinician, group care implementer
16	Nigeria	Yes	Clinician, group care implementer
17	Nigeria	Yes	Clinician, group care implementer
18	Nigeria	Yes	Clinician, group care implementer
19	Suriname	Yes	Researcher
20	United Kingdom	Yes	Clinician, group care implementer, researcher
21	United Kingdom	No	Clinician, group care implementer, researcher,
22	USA	Yes	Clinician, researcher
23	USA	Yes	Researcher
24	USA	Yes	Researcher
25	Zanzibar	Yes	Clinician, group care implementer

**Table 2:** Practice monitoring and reporting data - demonstrating the value of group care example template

Data to be collected	When	By Whom	For whom
ID of participants	At recruitment	Recruiter	Local provider Facilitators Health service
Demographics of participants (e.g., ethnicity, socioeconomic status, language support needs etc.)	At recruitment	Recruiter	Local provider Facilitators Funders
Mandated clinical data: Health Visitor pro-forma	All	Facilitators	Health service
Attendance (numbers attending each session)	All	Facilitators	Local provider Facilitators Funders
ID of facilitators	All	Facilitators	Local provider
Numbers of sessions	All	Facilitators	Local provider Funders
Facilitator’s reflection	All	Facilitators	Facilitators Local provider
National target: Vaccine uptake	All postnatal sessions	Facilitators	Funders Local provider
Feedback from participants	Multiple sessions	Participants & facilitators	Facilitators Service Users Local provider Funders Health service
WORKED EXAMPLE/From Parenting Circles in the UK (group care model carrying on an antenatal Pregnancy Circles for three postnatal sessions): Data collection form (implemented as normal care, funded by local commissioners; not in the context of research)

**Table 3:** Recommended research and evaluation outcomes and indicators

	Outcome	Indicator	Data source	Collection Methods/Suggested tools for measurement
1	Increased number of postpartum people having a postnatal contact within 2 weeks of delivery	# of postpartum people who had a postnatal visit within 2 weeks of delivery	Patient record/appointment book	Extract from health records
2	Increased rates of family planning use	# of postpartum people accepting family planning methods	Patient record/register of group care	Number of family planning methods dispensed/prescribed/inserted to women / direct ask of women/self-report
3	Increased rates of postpartum people with birth spacing (2–3 years)	# of people pregnant > = 2 years after birth of child	Patient record/register of group care	Extract from health records
4	Increased rates for postpartum people reporting they are following breastfeeding Infant and Young Child (IYCF) practices	# of postpartum people reporting they are following breastfeeding IYCF practices	Patient	Self-report
5	Increased number of children who receive all recommended vaccinations at the recommended timepoints	# of children who have completed their vaccinations according to local schedule by their second birthday	Patient record/registers	Extract from health records
6	Increased child development screenings	# of children provided with childhood development screenings	Patient record/register of group care	Extract from health records
7	Increased number of children completing all required/ suggested developmental screenings until age 2	# of children who have completed all childhood development screenings until age 2	Patient record/ register of group care	Extract from health records
8	Reduced number of children with malnutrition until age 2	# of children who have been diagnosed with malnutrition	Patient record/registers	Anthropometric measurements
9	Improved postnatal depression scores	# of postnatal people with improved depression scores	Validated screening tool	PHQ9 [26], EPDS [27], SRQ [28]
10	Improved postnatal anxiety outcomes	# of postnatal people with improved anxiety outcome	Validated screening tool	GAD7 [29]
11	Decreased number of postnatal people with anemia	# of postpartum people with anemia	Patient record	Extract from health records
12	Decreased number of postnatal people with hypertension	# of postpartum people with hypertension	Patient record	Extract from health records
13	Decreased number of postnatal people with diabetes	# of postpartum people diagnosed with Type 2 diabetes	Patient record	Extract from health records

### FRAME adaptations and modification guide

#### When was the modification made?

The blueprint was created in the pre-implementation phase to prepare sites to pilot the adapted CenteringParenting^®^ model.

#### Were adaptations planned?

The decision to modify CenteringParenting^®^ was planned and proactive. The main modifications are to increase focus on the maternal and infant/child dyad care, to maximize fit in diverse settings, and to help ensure implementation success before implementation.

#### Who participated in the decision to modify?

The four workshops held in Kenya had 20 participants from 13 countries. Subsequent synchronous virtual incubator sessions had a range of 5–15 people per session in attendance from 13 countries (see Table 1). All participants had experience with group antenatal care either as a clinician, group care implementer and/or researcher, and some participants had experience with group postnatal and/or well-child care. Participants had a range of experience from program leaders/managers to funders to intervention developers to researchers to practitioners and clinicians.

#### What is modified?

We modified the content and schedule of integrated care and made contextual modifications to the format and how the intervention is evaluated. The model is designed to be a continuation of group antenatal care so that groups of postnatal people and infant/child dyads and their family members (when appropriate) can continue to receive care together for the first two years after birth. It can also be implemented for new cohorts of families after birth starting at 1–2 weeks after birth depending on country context, health system capacity, and the postnatal person’s preferences. Participants reported that evaluation of the model should include international targets such as immunizations and maternal mental health, local priorities, fidelity measures, satisfaction and experience, and empowerment of families.

#### At what level of delivery were the modifications made?

The modifications were made at the individual level in terms of adding clinical assessments of the postnatal person which would impact both the individual attending group care and practitioners implementing group care. The modifications also emphasize family-centered care, with a specific focus on providing dyad care to both the postnatal person and their child. These modifications would in turn impact the clinic/organization in terms of selecting providers that can care for both the postnatal person and child as well as ensuring that suggested health assessments and services can be delivered within the group space. Participants hypothesized that this modification would impact the larger network and community with a focus on families and integrated health care services and lead to more efficient health care service delivery and improved outcomes.

#### Content modification

Elements were added to focus on the integration of care for the postnatal person. The packaging of materials was modified to create a clear emphasis on care of the postnatal person. In addition, participants lengthened exposure to the model, which traditionally starts in some places at six weeks after birth to create a model of group care that can start at one-two weeks after birth and extend through two years after birth. Participants discussed at length the challenges that many countries and contexts may face implementing group care before six weeks after birth when in some cultures and contexts postnatal people do not leave their home for the first six weeks or may not feel comfortable traveling to the clinic. However, participants reached consensus to create an “ideal model” that, when implemented should be adapted to each setting to meet the needs of postnatal people as well as be realistic to implement within each culture and context (e.g., start group care at six weeks after birth instead of 1–2 weeks after birth).

**Fig. 2:**
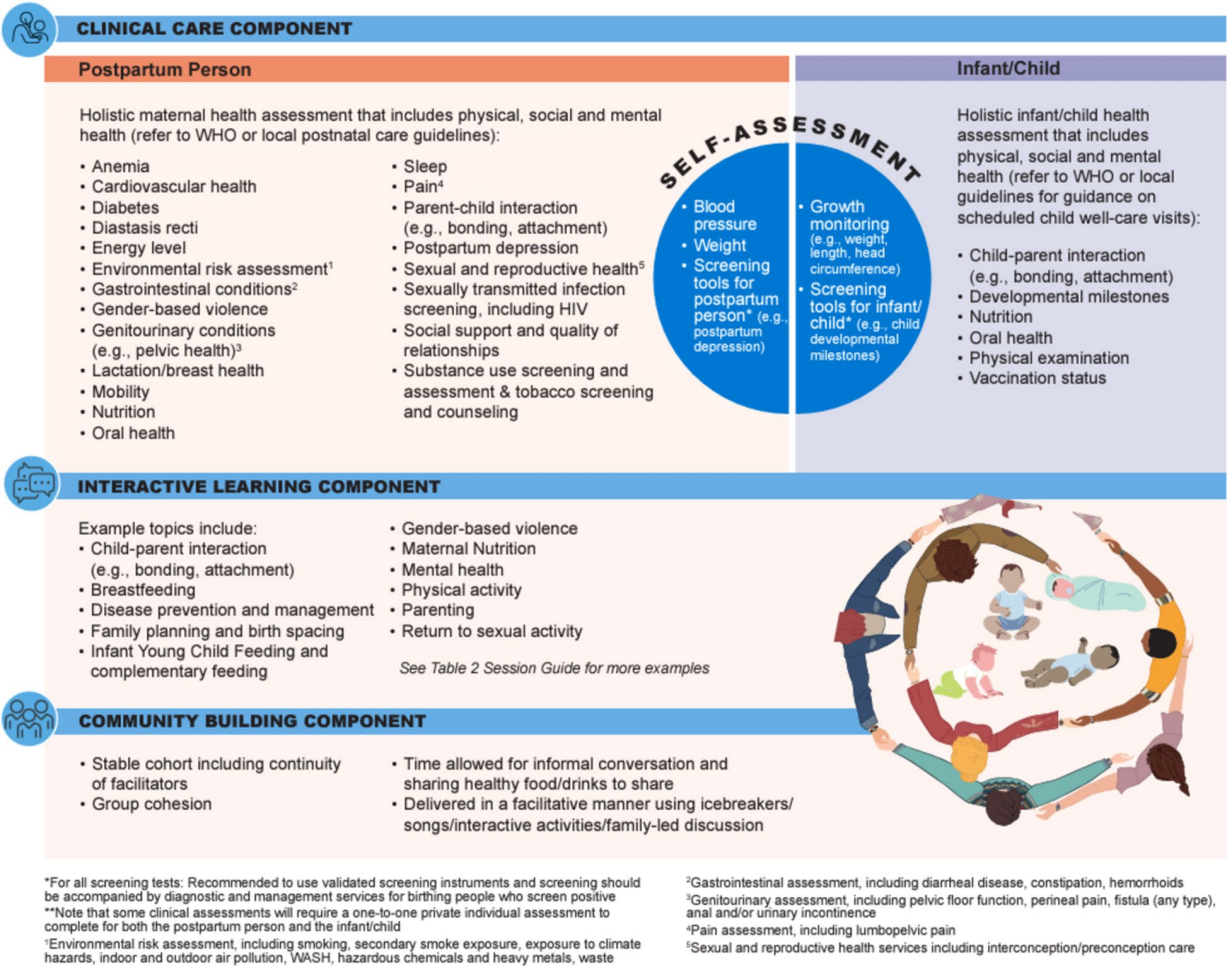
Guide for health assessments, interactive learning and community building applicable at all sessions

Figure 2 provides the blueprint details with a description of recommended clinical assessments, interactive learning and community building that can be applicable at all sessions but may not be possible to cover entirely in one session. This session guide is a result of the free listing, pile sorting, and ranking process. Participants worked together sharing their experiences from different countries and contexts to make this blueprint globally applicable with a focus on maternal and infant/child dyad care.

Participants took into consideration available evidence related to maternal and infant/child health when making decisions on what to prioritize to include in the model. For example, many participants reviewed literature related to maternal and infant/child morbidities and mortality patterns globally as well as common complaints reported in clinical care/practice to ensure that what is included is well informed based on the latest evidence-based practice. Participants used the *WHO recommendations on maternal and newborn care for a positive postnatal experience* [30] to guide recommendations for clinical care through the first six weeks after birth and the WHO’s *Improving the health and wellbeing of children and adolescents: guidance on scheduled child and adolescent well-care visits* [31] to guide recommendations for clinical care for the infant/child as well as families up through the first 1000 days. Participants also discussed how it will be critical for health care workers to also refer to their local guidelines to guide the clinical care they deliver within each context that group care is being implemented. Participants also discussed the specific clinical assessments that could be considered for the postnatal person at specific timepoints that might not occur at every visit and for which there is currently no standard of care globally beyond six weeks after birth. For example, screening for endocrine/metabolic conditions beyond six weeks after birth, HIV testing (context specific), tuberculosis screening (context specific), and oral health were added. Additionally, focusing on transitioning the postpartum person to primary care in the one to two years after birth will ensure that their health care needs are met, to prevent any negative consequences of labor or childbirth from developing into a chronic condition.

Participants discussed how there will be challenges to implementation depending on the health care system context in which it is delivered. Participants also discussed how some of the recommended health assessments may need to be done individually, particularly with an emphasis on the early postnatal visits, and that an individual postnatal intake might be necessary for both the postnatal person and the infant.

**Fig. 3:**
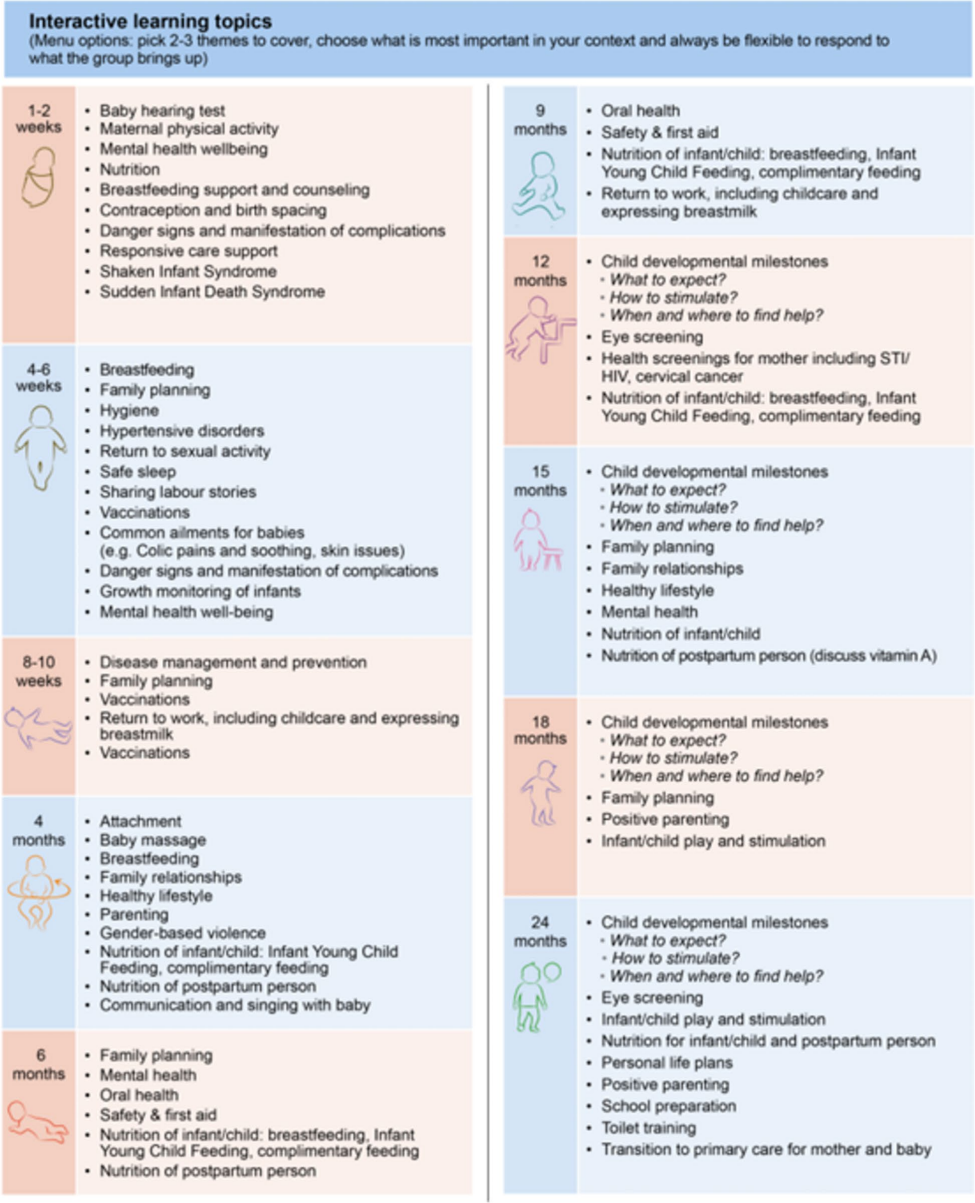
Session guide for interactive learning topics at recommended timepoints

Figure 3 summarizes the suggested interactive learning topics by timepoint, noting that what is discussed should be flexible and responsive to meet the needs of the group.

#### Evaluation modification

Participants discussed two different types of evaluation: one for research purposes and the other for quality improvement in clinical practice. When done in practice, participants felt it was important to reframe evaluation as a process of regularly scheduled reporting that elucidates the value of group care as well as what is working well and what needs to be addressed and improved rather than labeling the process as “evaluation” per se, which can have negative connotations to those providing care.

To demonstrate the value of group care in practice, it is important to take a team-based approach so that the team can assess what is working and what is not and make needed adjustments. A template can be used to collect data that will be beneficial for improving practice over time. Participants discussed how it is important from the beginning to designate who on the clinic team will be collecting, analyzing, and disseminating data so that clear processes are developed. Table 2 is a template for data collection to demonstrate the value of group care in practice. This template can be adapted for local contexts to ensure that data collected aligns with national health care outcome targets and agreed upon indicators for each setting.

Participants recommended not collecting data on all the topics covered in the sessions, other than mandated clinical care data by each health care system. The rationale for this was that the model is meant to be participant-led so that if there are other topics that participants want to discuss, there is flexibility to do so. If one is ticking off the topics on a rigidly defined plan, the valuable free-flowing discussions that should emerge from the group care model will not take place. Participants cautioned to not let data collection take precedence over care, information-sharing, and relationship-building.

Participants discussed how it was important for group care facilitators to be co-designers of the evaluation so that they know if the service they are providing is running efficiently and effectively. Participants suggested using a self-evaluation tool for each visit so that facilitators are not only evaluated by external actors but also take part in reflecting on their fidelity to the model, how facilitative they were, and how involved group members were (see Additional File 1) for example self-reflection template for group care facilitators). Participants also discussed the importance of how postnatal people, and their families should be a part of the evaluation so that their voices are heard as a part of the evaluation and the intervention is responsive to their needs, desires and satisfaction and in addition for prospective families to understand the value of group care and its impact on outcomes.

Participants discussed that evaluation in the context of research will be more detailed and will be collected in partnership with researchers within health systems or academic institutions, and professional associations for the purposes of building the evidence base for this adapted model.

Table 3 outlines suggested research and evaluation outcomes, indicators, data sources, and collection methods. When discussing outcomes and indicators participants discussed the importance of aligning these with research methods. For example, depending on the research question and methodology this will dictate what types of comparisons will be made to determine whether there are improvements in suggested outcomes and indicators (e.g., international, national or facility level comparisons).

#### Relationship to fidelity

All modifications made throughout this process were fidelity-consistent and preserved the core components of group care for it to be effective. While not the focus of the incubator sessions, participants discussed how training is integral to maintaining model fidelity. Participants discussed how training is necessary to be able to deliver group care effectively and in line with the core components. This is particularly important because group care is a very different approach to traditional health care delivery which often relies on didactive information-giving versus facilitated discussion and interactive learning which is a core pillar of group care.

#### Goals and rationale for the modifications made

The goals of the adaptation included: to increase the reach and retention of participants in group care beyond birth; improve feasibility so that this blueprint can be used across diverse settings; improve fit with recipients; increase satisfaction with its delivery and address cultural factors; and improve effectiveness and outcomes to improve maternal and child health through group care.

The reasons for adaptation included socio-political factors, to address inequities in health care delivery, and to create a model that addresses the gap in global postnatal care guidelines that focus only up to six weeks after birth; no guidelines exist beyond that. These modifications provide a blueprint for providing care for both the postnatal person and infant/child throughout the entire first two years after birth. In addition, at the provider level, participants discussed how providers can support these modifications to integrate care services and provide family-centred care. And at the recipient level these modifications were made to optimize group care for the dyad to improve maternal and child health outcomes.

## Discussion

The study successfully developed a blueprint for group care beyond birth, retaining the core components of health assessment, interactive learning, and community building from the original CenteringParenting^®^ model and systematically reports the adaptations and modifications made with the use of the FRAME. Advancing the science of adaptation of evidence-based interventions is key for addressing inequities in healthcare delivery to ensure that models of care can be updated to meet the needs of families and health care systems across settings [32]. Using frameworks to conduct the adaptations systematically is recommended to identify optimal adaptation across different contexts and populations [32]. The use of human-centered design and rapid qualitative analysis allowed for a collaborative approach to developing this blueprint for group care beyond birth. The inclusivity of a large and diverse set of global perspectives to co-create this blueprint ensures that it is sensitive and contextually adaptable to different settings. Collectively, this process resulted in a blueprint that increases the likelihood for scalable implementation across health systems globally.

This study addresses a significant gap in maternal health globally by recognizing the neglected needs of both the postnatal person and the infant/child beyond six weeks after birth. This study addresses the need for extended, holistic postnatal care after birth by adapting the CenteringParenting model which was initially designed as a dyad model but in practice has primarily focused on the infant/child, our adaptation aims to realize the model’s full potential by providing comprehensive integrated postnatal and pediatric care (dyad care) [2]. Key adaptations included a focus on maternal health and early engagement post-birth, accommodating varying cultural practices and health system capabilities. The inclusion of comprehensive health assessments and integration of social needs into care delivery represents a much-needed shift towards holistic maternal and infant health and is responsive to the growing evidence base that there are neglected medium and long-term consequences of labor and childbirth globally and postnatal people have ongoing care needs that extend beyond the traditional six-week postnatal period [2, 15]. For example, non-communicable diseases (NCDs) are increasingly being recognized as an important cause of maternal morbidity and mortality [33]. The blueprint reflects the importance of early identification and management of NCDs in maternal health care such as cardiovascular health, mental health, and diabetes recommended to be addressed throughout all the group care beyond birth visits. Research suggests that perinatal care measures are incomplete without assessing the dyad and that maternal and infant/child health outcomes are linked [34]. The blueprint presents the foundation for providing dyadic care to both the postnatal person and infant/child and support the health of the dyad and their families.

Through this work, we not only produced a foundational blueprint, but also showed a systematic way of documenting the necessary adaptations or modifications that may be needed on a local level so that these adaptations can be well-described and justified. The blueprint also includes suggested evaluation metrics, encouraging continuous quality improvement to evidence-based practices, thereby contributing to an ever-evolving and responsive group care model. Evaluation indicators were identified with implications for both research and practice and to capture outcomes that highlight the model’s potential effectiveness and for quality improvement purposes. Future research is recommended to test the blueprint using the recommended outcomes and indicators. Standardizing evaluation of the model across diverse settings will help build the evidence base for this promising health care model to improve maternal and infant/child outcomes.

### Limitations and potential challenges

The adaptation process can be further optimized by including the target population as participants in this process. Challenges remain with health systems that silo maternal and infant/child health services and have separate providers and locations for postnatal and well-child care services. When considering implementation, it will be crucial to identify skilled professionals and a team-based approach to deliver this integrated maternal and child health care service delivery model so that services can be provided to both the postnatal person and the infant/child. This might require some initial professional training and willingness and capacity of institutions to allocate health care providers to this model of care that are cross trained in postnatal and pediatric care. Some settings have used interdisciplinary teams to deliver group care so that services can be delivered to the dyad [35, 36]. Another challenge is ensuring that individual care needs are met when needed, particularly in the early postnatal period. In some settings group care is implemented in tandem with home visiting in the first six weeks to provide both individual and group care [37]. Adding services in addition to group care may be helpful especially in contexts where group care may not be possible before six weeks after birth and there are care needs that need to be addressed individually. In addition, adding home visits for group participants with needs not met by the group is another potential strategy to provide increased support for populations such as young adolescents or those with high social needs. Opportunities also exist to bring in further innovation like using technology to bolster social connection and community building.

## Conclusions

Adaptations and modifications of evidence-based models and practices are important to enhance their impact across settings. This study demonstrates the utility of the FRAME as tool for systemically describing and reporting the adaptation process and how it leads to the final blueprint. Co-designing an adapted Centering-based group care model that integrates both postnatal and pediatric care fills a significant gap in postnatal care delivery and fosters a more holistic, integrated approach which when implemented holds the promise of profoundly impacting maternal and child health care service delivery worldwide. This study sets the stage for future research to test this blueprint across different settings using standardized evaluation outcomes and indicators. The use of such evaluations is crucial for building a robust evidence base for this innovative group healthcare model, aiming to improve maternal and infant health outcomes globally.

## Supplementary Information

Supplementary Material 1.

## Data Availability

The datasets during and/or analyzed during the current study available from the corresponding author on reasonable request.

## Abbreviations

COREQ: Consolidated criteria for reporting qualitative research
EPDS: Edinburgh Postnatal Depression Scale
FRAME: Framework for Reporting Adaptations and Modifications to Evidence-based interventions
GAD: Generalized Anxiety Disorder
HCD: Human-centered design
HIV: Human Immunodeficiency Virus
IYCF: Infant and Young Child
LMIC: Low- and middle-income country
MCH: Maternal and child health
NCD: Non-communicable disease
PHQ: Patient Health Questionnaire
SRQ: Self-Reporting Questionnaire
UK: United Kingdom
USA: United States of America
WHO: World Health Organization
