# Supplementary figures and images for: Integrating postnatal care into the redesign of group care beyond birth
Source: Arch Public Health. 2025 Feb 13;83:34. doi: 10.1186/s13690-025-01508-4 (PMC11823241; doi:10.1186/s13690-025-01508-4)

Additional File 1: Example self-reflection template for group care facilitators


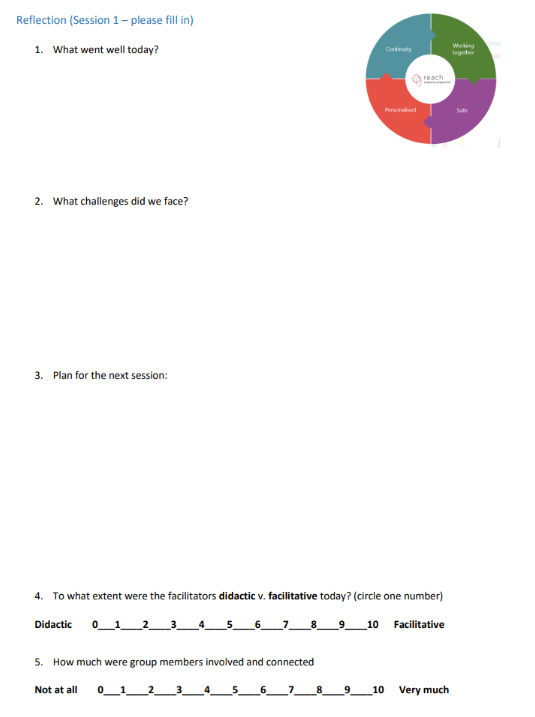

Supplement: Supplementary file 1 — Supplementary Material 1. [file 13690_2025_1508_MOESM1_ESM.docx]
